# Preparation and Encapsulation of DPP-IV Inhibitory Peptides: Challenges and Strategies for Functional Food Development

**DOI:** 10.3390/foods14091479

**Published:** 2025-04-24

**Authors:** Rui Zhao, Ye Zhou, Huifang Shen, Lijun Guan, Yao Wang, Xinting Shen, Fei Wang, Xinmiao Yao

**Affiliations:** 1 Food Processing Research Institute, Heilongjiang Academy of Agricultural Sciences, Harbin 150086, China; lilyamongthorns@163.com (R.Z.); zhouye614@163.com (Y.Z.); shenhuifang_1987@126.com (H.S.); guanlijun1983@gmail.com (L.G.); wang1221yao1221@163.com (Y.W.); 15663585599@163.com (X.S.); wangf2022822@163.com (F.W.); 2 Heilongjiang Province Key Laboratory of Food Processing, Harbin 150086, China; 3 Heilongjiang Province Engineering Research Center of Whole Grain Nutritious Food, Harbin 150086, China

**Keywords:** DPP-IV inhibitory peptide, bioinformatics, enzymatic hydrolysis, fermentation, encapsulation

## Abstract

Dipeptidyl peptidase IV (DPP-IV) inhibitory peptides have emerged as promising functional ingredients for managing type 2 diabetes due to their ability to enhance insulin secretion and improve glycemic control. This review provides a concise overview of current strategies for the preparation and encapsulation of DPP-IV inhibitory peptides, with a focus on food industry application, evaluating bioinformatics for substrate selection, and methods like mild enzymatic hydrolysis, cost-effective fermentation, and high-purity chemical synthesis for peptide production. Challenges associated with incorporating these peptides into food products are addressed, including impacts on sensory properties, stability during processing and digestion, and the need for effective delivery systems to enhance bioavailability. Potential solutions to improve peptide stability and targeted release, such as emulsions, liposomes, and nanoparticles, are explored. Future research directions are outlined, emphasizing the necessity for scalable production methods, co-encapsulation strategies, and consumer acceptance studies to facilitate the commercialization of DPP-IV inhibitory peptides as functional food ingredients. By addressing these key areas, this review aims to provide a theoretical foundation and practical guidance for the development of DPP-IV inhibitory peptides, paving the way for their broader application in the prevention and management of type 2 diabetes.

## 1. Introduction

Diabetes, recognized as a global health crisis, significantly impacts the well-being and quality of life of billions of people worldwide. DPP-IV is a serine protease that specifically cleaves glucagon-like peptide-1 (GLP-1) and glucose-dependent insulinotropic polypeptide, leading to the inactivation of these incretin hormones [1]. Therefore, inhibiting DPP-IV activity can extend the duration of action of these endogenous hormones, thereby enhancing glucose control. Incorporating these inhibitors into functional foods may offer health benefits, including better blood glucose management and a reduced risk of developing type 2 diabetes.

Although DPP-IV inhibitors, especially gliptin drugs, have demonstrated remarkable efficacy in the management of diabetes, their application might evoke a series of adverse reactions [2]. Consequently, researchers have shown great interest in DPP-IV inhibitory peptides derived from food proteins. These peptides, generally obtained through specific hydrolysis processes, demonstrate significant inhibitory effects on DPP-IV in biochemical assays, cell-based studies, animal experiments, and even human trials [3,4,5]. In comparison to many traditional chemically synthesized drugs, these food-derived peptides offer enhanced safety profiles and reduced side effects, rendering them more suitable for long-term therapeutic interventions [6]. Although their pharmacological efficacy generally falls short of that exhibited by synthetic drugs, their potential as functional food ingredients has gained widespread recognition.

Currently, remarkable progress has been achieved in the research on DPP-IV inhibitory peptides, laying a solid theoretical groundwork for their commercial application. However, when it comes to large-scale production and food application, numerous challenges remain to be overcome. These include their impact on food sensory perception, stability during food processing and digestion, and so on. This review centers on the domains directly related to the food application of DPP-IV inhibitory peptides, such as the selection of protein substrates, their preparation, and encapsulation. While acknowledging the importance of activity validation and mechanism research in this field and their critical role in guiding production practices, this review will not elaborate on these aspects in detail due to their inherent complexity, broad scope, and foundational nature for applied research. By systematically reviewing relevant research achievements and development trends and discussing challenges and corresponding solutions, this article aims to provide a practical reference for the application of DPP-IV inhibitory peptides in the food industry.

## 2. Strategies for Efficient Peptide Production

### 2.1. Selection of Protein Substrates for DPP-IV Inhibitory Peptides

**Figure 1:**
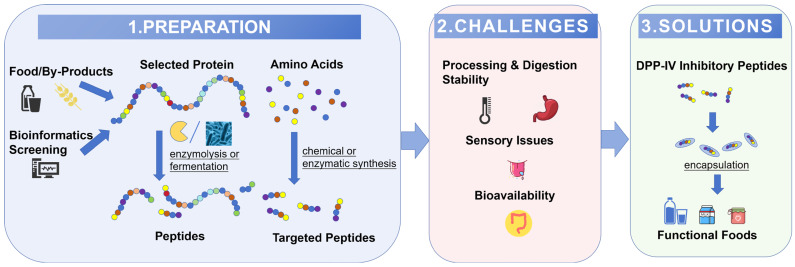
Schematic overview of the development pathway for DPP-IV inhibitory peptide-based functional foods.

As illustrated in Figure 1, the efficient production of DPP-IV inhibitory peptides constitutes an essential foundation for their application in food. Regarding enzymatic or fermentation approaches for preparing DPP-IV inhibitory peptides, the process initially involves the selection of appropriate protein substrates. The traditional research approaches rely on empirical in vitro experiments. Undeniably, this conventional method has been extensively applied in numerous experimental studies and has accumulated abundant knowledge in the past. However, these processes are typically time-consuming, labor-intensive, and of low efficiency. With the progression of bioinformatics, researchers can complete the work within a much shorter period, thereby screening and evaluating the potential substrates of DPP-IV inhibitory peptides more effectively. Additionally, the source, stability, and availability of parent proteins are also crucial factors that ought to be considered in the selection procedure.

Therefore, this study aims to illuminate the current research trends: the application of bioinformatics methods capable of enhancing research efficiency and the utilization of bulk agricultural by-products conducive to achieving sustainable resource utilization.

#### 2.1.1. Bioinformatics in DPP-IV Inhibitory Peptides Substrate Evaluation

The parental protein’s amino acid sequence greatly affects the quality and quantity of yielded DPP-IV inhibitory peptides. Bioinformatics provides a scientific and efficient means to rapidly evaluate the potential of proteins in generating DPP-IV inhibitory peptides. By analyzing the occurrence frequency of known DPP-IV inhibitory peptides in proteins and their activity, one can predict a protein substrate’s potential [7]. Although this approach does not consider whether every peptide can be effectively cleaved, it can initially determine the maximum potential of proteins for DPP-IV inhibitory peptide preparation and is suitable for initial screening among many candidate proteins.

In contrast, in silico simulation of proteolysis can not only predict the peptide segments generated by a single protease but also compare the enzymatic hydrolysis effects of different proteases as well as their combinations (e.g., using tools such as BIOPEP, PeptideCutter, and ProtParam). This offers guidance for the selection of suitable proteases and the optimal protein–protease combination in actual proteolysis. For instance, Li et al. [8] performed simulated hydrolysis of hazelnut protein using five proteases or their combinations. It was found that the combination of chymotrypsin and papain exhibited the most superior effect, generating the largest number of active fragments of DPP-IV inhibitory peptides. Combination enzymatic hydrolysis can generate new inhibitory peptides while retaining those produced by chymotrypsin. Likewise, Han [9] employed the BIOPEP to simulate the hydrolysis processes of ten oilseed proteins by alcalase and pepsin. The results indicated that napin treated with pepsin (pH > 2.0) exhibited the highest potential activity.

Moreover, to learn more about the activity, affinity with DPP-IV, and mode of action of the obtained peptides, further studies could be carried out through methods such as quantitative structure–activity relationships, molecular docking, and deep learning [10,11]. Some excellent reviews discussed these aspects comprehensively [7,12], and they will not be elaborated on in detail here. The efficient DPP-IV inhibitory peptide verified experimentally can be applied to peptide synthesis.

Due to the complexity of the advanced structure of proteins and the influence of enzymatic hydrolysis conditions, it is challenging to guarantee that all predicted cleavage sites can be effectively cleaved during the actual enzymatic hydrolysis process. Therefore, notable discrepancies between actual hydrolysis and predictions are often observed [12]. For instance, despite the prediction that 28 DPP-IV inhibitory peptides would be released in silico, only 18 were actually identified from the hydrolysate of α-Lactalbumin [13]. To overcome these constraints, integrating actual enzymatic hydrolysis with bioinformatics methods provides a more reliable approach. By focusing on the peptides obtained from actual hydrolysates and leveraging bioinformatics tools for in-depth analysis, researchers can rapidly identify high-potential peptide sequences.

To illustrate this integrated approach, Zhu et al. [14] grounded their research in the optimal results derived from computer simulations and prepared eggshell membrane hydrolysates using a dual-enzyme hydrolysis method. Through nano LC–MS/MS, they identified three high-abundance DPP-IV inhibitory peptides from the hydrolysate (<3 kDa). Moreover, molecular docking was utilized to investigate the affinity of these peptide sequences to DPP-IV, and it was discovered that YPEPPPQ is the most promising DPP-IV inhibitory peptide, with an IC_50_ value of 1.67 ± 0.02 mM [14].

Bioinformatics has greatly reduced the time, economic, and labor costs of laboratory studies on DPP-IV inhibitory peptides, expediting the research progress. Nevertheless, considering the limitations of bioinformatics, it is necessary to experimentally validate the predicted results.

#### 2.1.2. Utilizing Protein-Rich Processing By-Products as Substrates

Besides bioinformatics evaluations, researchers also pay close attention to the utilization of high-protein by-products from agriculture, livestock processing, and fishery as stable sources for the preparation of DPP-IV inhibitory peptides. These by-products not only lower production costs but also optimize resource utilization, which holds significant importance in addressing resource scarcity and environmental pressure.

Collagen in animal skin is abundant in Pro, which is highly prevalent in efficient DPP-IV inhibitory peptides [15,16]. This makes animal skin an outstanding source of DPP-IV inhibitory peptides [17,18,19]. Wang et al. (2021) [19] obtained a DPP-IV inhibitory peptide GPAGPOGFPG from the enzymatic hydrolysate of sheep skin, with an IC_50_ value of 67.12 ± 0.64 μM. Additionally, whey protein, a by-product of cheese processing, is also an excellent source for the production of DPP-IV inhibitory peptides. For example, Nongonierma et al. [20] isolated VPV from the hydrolysate of camel whey protein concentrate with an IC_50_ value of 6.6 ± 0.5 µM for DPP-IV inhibitory activity [20]. It has also been reported that animal blood, eggshell membranes, etc., have been utilized for the preparation of DPP-IV inhibitory peptides [14,21].

A variety of agro-industrial by-products derived from plants are also good sources of DPP-IV inhibitory peptides. The by-products of oilseed crops are rich in proteins and can be employed for the preparation of DPP-IV inhibitory peptides, such as olive seeds, black sesame cakes, hazelnut meal, etc. [22,23,24,25]. For example, Kong et al. [24] obtained the most potent active peptide MRPDEDEQEGQ from the hydrolysate of defatted walnut, and its inhibitory activity against DPP-IV was 76.19% at 0.25 mg/mL. Moreover, the utilization of other protein-rich bulk by-products has also been reported, such as corn distillers’ solubles [26] and rice bran [27].

### 2.2. The Preparation of DPP-IV Inhibitory Peptides

Although some food processing procedures can generate DPP-IV inhibitory peptides [6], these methods often lack specificity and have limited yields. To prepare DPP-IV inhibitory peptides effectively, researchers have developed the following strategies: enzymatic hydrolysis or fermentation for top–down degradation of proteins; and chemical or enzymatic methods for the bottom–up construction of peptide sequences from amino acids [28], as shown in Table 1. Until now, various DPP-IV inhibitory peptides have been prepared from diverse food proteins. These peptides exhibit remarkable diversity in terms of origin, sequence, length, and activity. Detailed information can be found in Table 2.

#### 2.2.1. Enzymatic Hydrolysis

Enzymatic hydrolysis, which serves as an effective means of preparing DPP-IV inhibitory peptides, releases various peptide segments by protease cleavage of peptide bonds at specific sites. Once the protein substrate is identified, specific pretreatment is typically required. Subsequently, appropriate proteases should be selected and specific enzymatic parameters optimized, leading to the production of a hydrolysate with DPP-IV inhibitory activity.

Appropriate pretreatment steps exert a remarkable promoting impact on the enzymatic hydrolysis process, especially when enzymatic hydrolysis is conducted using processing by-products. A variety of impurities within the raw materials may interfere with the enzymatic hydrolysis process, necessitating pretreatment to ensure optimal conditions for enzyme activity. For example, organic solvents are frequently employed to extract oil from hazelnut meal [39], and alkaline solutions are commonly utilized to soak collagen-rich animal by-products to eliminate impurities such as fat [16,19].

Furthermore, appropriate treatment can disrupt the advanced structure of proteins and expose the enzymatic cleavage sites, thereby enhancing the efficiency of enzymatic hydrolysis and the bioactivity of the products [40]. Common treatment methods include thermal processing, extrusion, microwave, and non-thermal processing technologies (such as ultrasound, hydrostatic pressure, and pulsed electric field) [41,42,43]. For example, after undergoing ultrasound treatment, the secondary structure of camel milk proteins changed, resulting in a noticeable increase in surface hydrophobicity and degree of protein hydrolysis. Additionally, the enzymatic hydrolysate reached its peak DPP-IV inhibitory activity after 30 min of ultrasound treatment [32].

Additionally, the combined use of various pretreatment methods can offer further benefits. For example, when ionic liquids and ultrasound were used together to pretreat cowhide collagen, there was a significant increase in DPP-IV inhibitory activity and the production of peptides with improved stability and higher yields of low molecular weight (MW) peptides under simulated gastrointestinal digestion (SGID) compared to using only one method [33].

Pretreatment is typically employed prior to enzymatic hydrolysis and sometimes during the enzymatic hydrolysis process to play an auxiliary role. For instance, microwave treatment was carried out during the Alcalase hydrolysis of cricket protein, which could enhance the degree of hydrolysis and decrease the IC_50_ value [44].

It is important to note that the impact of pretreatment on enzymatic hydrolysis is closely linked to the type of protease used. Different proteases have specific cleavage sites. When the same pretreatment is applied, it causes the same structural changes in proteins. However, due to the differences in protease cleavage site specificities, these identical protein structure changes can have varying effects on the hydrolysis efficiency of different proteases. For example, aqueous heating treatment significantly inhibited the hydrolysis of wheat protein by ProteAXH and Acid protease, but it promoted hydrolysis by Neutral protease and Flavourzyme (*p* < 0.05) [45]. Therefore, it is necessary to determine the protease species first and then optimize the pretreatment conditions.

The species of protease plays a vital role in influencing the outcome of enzymatic hydrolysis. Among the proteases commonly employed for the enzymatic preparation of DPP-IV inhibitory peptides are several categories, including animal-derived digestive enzymes, plant-derived proteases such as papain, bromelain, and ginger protease, as well as microbial enzymes like Alcalase and Flavourzyme. Different proteins possess unique amino acid sequences and three-dimensional structures, resulting in varied sensitivities to proteases. The specificity of protease cleavage sites dictates the efficiency of hydrolysis on particular protein substrates. The specificity of its cleavage sites, in conjunction with the amino acid sequence composition of the protein substrate and the specific enzymatic conditions, jointly determines the peptide sequence generated. To prepare DPP-IV inhibitory peptides more effectively, researchers have developed various enzymatic hydrolysis strategies.

The species of protease significantly influences the outcome of enzymatic hydrolysis. Different proteins, due to their unique amino acid sequences and three-dimensional structures, show varied sensitivities to specific proteases. Protease specificity at cleavage sites, along with substrate composition and reaction conditions, determines the resulting peptide sequence. For example, to efficiently prepare DPP-IV inhibitory peptides, researchers have developed various enzymatic hydrolysis strategies.

Considering the distinct substrate specificities of different proteases, selecting enzymes based on protein structure is essential for maximizing DPP-IV inhibitory activity. Since the hydrolysis sites of a single protease are often limited, many studies have utilized combinations of various proteases to achieve complex enzymatic hydrolysis. Particularly, the combination of endopeptidase and exopeptidase has been widely adopted. For instance, using Proteinase K and α-chymotrypsin together significantly enhances the DPP-IV inhibitory activity of Bactrian camel milk protein compared to single-enzyme hydrolysis. This demonstrates a synergistic effect between these two proteases, leading to improved hydrolysis efficiency and enhanced DPP-IV inhibitory activity [32].

Furthermore, considering that peptide segments may be further degraded by digestive enzymes during the digestion process, enzymatic hydrolysis using digestive enzymes has unique advantages, as peptides prepared in this manner may have stronger digestive stability [20].

Strategically designed enzymatic approaches are employed to efficiently liberate Xaa-Pro (XP)-type peptides with DPP-IV inhibitory activity. Studies have demonstrated that oligopeptides with an N-terminal amino acid sequence of XP exhibit potent inhibitory activity against DPP-IV [46]. For instance, Taga et al. [47] implemented a single-enzyme strategy by utilizing ginger protease’s unique specificity for Pro at the P_2_ position, combined with substrate selection by choosing Pro-rich wheat protein. This approach yielded tripeptides such as QPQ, QPG, and QPF (IC_50_ values range from 56.7 to 79.8 µM), demonstrating high inhibitory activity against DPP-IV. Conversely, Wang et al. [48] developed a dual-enzyme synergistic system where papain preferentially hydrolyzes long XP-type peptides in initial stages, while ProtexA subsequently processes short XP-type fragments. The synergy between these two enzymes, as demonstrated by experimental results, accelerates the extensive liberation of short XP-type peptides.

The optimization of enzymatic conditions is a crucial step for obtaining highly active DPP-IV inhibitory peptides. In addition to the types of proteases, factors such as the dosage of added proteases, substrate concentration, enzymatic hydrolysis temperature, pH value of the system, and the duration of enzymatic hydrolysis all impact the effectiveness of the process. To optimize these factors, it is generally necessary to conduct single-factor experiments in combination with orthogonal or response surface methodology experiments [13,49].

The enzymatic hydrolysis for preparing DPP-IV inhibitory peptides has significant advantages. Firstly, it operates under mild conditions without the requirement for toxic and harmful organic solvents and catalysts. The process is relatively clean and environmentally friendly. Secondly, the enzymatic hydrolysis efficiency is high, enabling the acquisition of the target product within a relatively short period and facilitating large-scale production. Finally, the enzymatic method has shown excellent controllability. By meticulously selecting specific proteases and optimizing the conditions for enzymatic hydrolysis, it is possible to precisely control the length and sequence of the peptide chain, thereby obtaining DPP-IV inhibitory peptides with high activity.

However, enzymatic hydrolysis is also subject to certain limitations. Firstly, the cost of proteases is relatively high, especially when high purity or special properties are required. The application of immobilized enzyme technology can enhance the reuse rate of proteases and contribute to reducing overall costs [50]. However, the immobilization process itself may require additional technical and cost investments. Secondly, strict control of enzymatic hydrolysis conditions and raw material quality is crucial. Different batches of proteases may vary in purity and activity, and minor fluctuations in enzymatic hydrolysis conditions may cause significant differences in the quality of the final product [51]. Thirdly, constrained by the cleavage sites of proteases and the spatial structure of proteins, it is difficult to completely release the potential DPP-IV inhibitory peptides from proteins. Additionally, in many instances, the DPP-IV inhibitory peptides prepared by enzymatic hydrolysis possess a distinct bitter taste, which severely restricts their application in food.

#### 2.2.2. Fermentation Technique

The generation of DPP-IV inhibitory peptides through fermentation involves the hydrolysis of proteins into small peptide segments by a series of proteases and peptidases produced by microorganisms. If raw materials hold proteins and a variety of other nutrients while lacking microorganism growth inhibitors, fermentation is favored for DPP-IV inhibitory peptide preparation. Herein, microorganisms can utilize environmental nutrients for growth and yield a rich enzyme system, including various proteases and peptidases. These enzymes not only efficiently hydrolyze proteins into bioactive peptides but also, due to the influence of microbial metabolic activities, endow fermented products with richer and milder flavor characteristics compared to those obtained through simple enzymatic hydrolysis. Moreover, the fermentation process generates additional beneficial components such as organic acids and vitamins, further enhancing the nutritional value and functionality of the final product.

DPP-IV inhibitory peptides have been identified from a multitude of fermentation products. For example, Olvera-Rosales et al. [52] introduced two probiotics, *Lactobacillus rhamnosus* GG and *Streptococcus thermophilus* SY-102, to co-ferment a whey solution. They found that the peptide segments with MWs below 2 kDa exhibited a DPP-IV inhibition rate exceeding 50%. In another investigation, Yang et al. [53] identified 125 DPP-IV inhibitory peptide sequences composed of ten or fewer amino acid residues from fermented four-day fermented fish (*Chouguiyu*). Among them, the IC_50_ values of four highly efficient DPP-IV inhibitory peptides selected by molecular docking ranged between 0.10 and 8.51 mM. Additionally, Mudgil et al. [37] found that probiotic-fermented milk from different livestock, such as cows, camels, and goats, displayed significant DPP-IV inhibitory activity. The IC_50_ values for fermented cow, camel, and goat milk were 0.17, 0.12, and 0.25 µg/mL protein equivalent, respectively. The above-mentioned studies indicate that fermentation is an effective way to obtain DPP-IV inhibitory peptides.

The fermentation and enzymatic hydrolysis can also work synergistically. During fermentation, microorganisms secrete various proteases (such as endopeptidases and exopeptidases) that can hydrolyze macromolecular proteins. Specific exogenous enzymes can also be added to supplement specific cleavage sites and generate target peptides in a targeted manner. Compared with single enzymatic hydrolysis or fermentation, this approach can reduce production costs and improve process efficiency. The synergistic method of fermentation–enzymatic hydrolysis has been widely used in the preparation of other bioactive peptides, but it has rarely been reported in the preparation of DPP-IV inhibitory peptides. Shen et al. [54] treated soybean meal using both fermentation and enzymatic hydrolysis methods. In terms of crude protein content, acid-soluble protein content, organic acid production, and oligosaccharide degradation, this combined treatment was significantly better than single fermentation or enzymatic hydrolysis (*p* < 0.05). Through peptidomic analysis combined with bioinformatics prediction, the small peptides produced after treatment have the potential for DPP-IV inhibition. However, this activity has not yet been experimentally verified. This combination of fermentation and enzymatic hydrolysis provides a new idea for improving the production of DPP-IV inhibitory peptides.

Despite fermentation being a crucial method for obtaining DPP-IV inhibitory peptides, considering the complexity and diversity of fermentation systems, directly utilizing unpurified fermented products presents a more cost-effective approach. This strategy avoids complicated purification steps while ensuring the retention of all naturally occurring bioactive substances, thereby providing comprehensive health benefits. Natto, a traditional Japanese fermented food made from soybeans, exemplifies this approach. In addition to DPP-IV inhibitory peptides, natto contains a variety of other active compounds, such as nattokinase, isoflavones, vitamin K2, phenolic compounds, γ-aminobutyric acid, and dietary fiber [55,56]. Therefore, consuming natto directly rather than extracting single bioactive peptides allows for the full utilization of its overall nutritional value, providing a more holistic health effect. This integrative usage simplifies processing and ensures that all potential functional components are available to consumers.

The superiority of the fermentation process is manifested in cost-effectiveness, as the fermentation microorganisms can synthesize the required proteases and peptidases during their proliferation, eliminating the need for expensive commercial enzyme preparations. Moreover, the multiple proteases and peptidases produced by the fermentation microorganisms interact synergistically, enriching and complicating the flavor of the fermentation products. Moreover, as mentioned above, due to the complexity of the system, a variety of bioactive substances may be present in the fermentation products, which could have synergistic effects to enhance their biological efficacy.

However, there are certain limitations in the fermentation-based preparation of DPP-IV inhibitory peptides. To begin with, the fermentation period is typically lengthy, resulting in relatively low production efficiency. Secondly, the pre-screening process for starter strains capable of high-efficiency production of DPP-IV inhibitory peptides is a labor-intensive and time-consuming task. Additionally, considering the diversity of components in the fermentation matrix, the purity of the obtained DPP-IV inhibitory peptides may not be optimal, and subsequent purification steps can be costly. Furthermore, there are issues such as poor enzymatic specificity and low yield of target peptides during fermentation [57]. The safety issues of fermentation cannot be ignored, as microorganisms may generate harmful secondary metabolites under certain conditions. Therefore, if fermentation is employed to prepare DPP-IV inhibitory peptides, appropriate starter strains should be selected, and the fermentation conditions and extent should be stringently controlled to ensure the safety and reliability of the product.

#### 2.2.3. Peptide Synthesis: A Tool for Validation and Specialized Production

Undoubtedly, the optimal approach to preparing high-purity specific DPP-IV inhibitory peptide sequences is through peptide synthesis. Chemical synthesis, particularly Fmoc solid-phase synthesis, plays a critical role in the laboratory validation of specific sequence activity and the investigation of structure–activity relationships [58]. For instance, Guan et al. [46] prepared a large number of tripeptide mixtures, Val-Pro-Xaa and Ile-Pro-Xaa, with DPP-IV inhibitory potential through chemical synthesis. After in vitro experimental verification, three peptides (VPV, VPI, and IPI) were shown to exhibit a strong inhibitory effect on human DPP-IV.

Given the limitations in applying chemical synthesis to food production, such as high cost, synthetic origin, and consumer preference, the enzymatic synthesis of DPP-IV inhibitory peptides using food-grade raw materials has gained attention in recent years. For instance, Yang et al. [59] utilized glutaminase derived from *Bacillus subtilis* to synthesize various γ-glutamyl dipeptides. Among them, γ-Glu-Met exhibited remarkable inhibitory activity against DPP-IV, with an IC_50_ value of 2.11 mM. Additionally, it offered multiple advantages, such as facilitating amino acid absorption and enhancing intestinal health. Furthermore, γ-glutamyl peptides possess higher digestive stability and easier permeability through cell membranes, resulting in improved bioavailability. All the materials used in the entire synthesis process are natural and comply with food-grade standards without relying on high temperature, high pressure, or organic solvents, thus making it environmentally friendly. In contrast to chemical synthesis, enzymatic synthesis holds greater superiority in terms of cost-effectiveness and environmental protection and is highly suitable for industrial production [59]. Therefore, the enzymatic synthesis of DPP-IV inhibitory peptides has great potential for industrial production.

Additionally, utilizing gene recombination technology to express specific peptide sequences in microorganisms represents a feasible approach for preparation. Although there are few reports on its application in the DPP-IV inhibitory peptide preparation, this technique has been successfully employed in the production of insulin, antibacterial peptides, hormones, growth factors, and so on [60,61].

Overall, peptide synthesis techniques, especially enzymatic synthesis, offer efficient and flexible solutions for the preparation of DPP-IV inhibitory peptides. To begin with, peptide synthesis is capable of generating target peptide sequences of high purity, which are typically present in a relatively low proportion in the entire enzymatic hydrolysate or fermented products. Secondly, highly efficient DPP-IV inhibitory peptides are typically oligopeptides, which raises the complexity of enzymatic hydrolysis and fermentation but reduces the difficulty of synthesis. It is also worth mentioning that amino acid substitutions, as well as modifications like phosphorylation and acetylation, which can be introduced during synthesis, can enhance peptide stability, biological activity, and bioavailability, thereby achieving improved efficacy [62,63].

In summary, enzymatic hydrolysis, fermentation, and enzymatic synthesis provide diverse approaches for the preparation of DPP-IV inhibitory peptides, each with its own advantages and limitations, as shown in Table 1. While chemical synthesis plays a critical role in validating peptide sequences and producing specific high-purity peptides, its application in functional foods is limited due to cost, regulatory hurdles, and consumer preferences for natural ingredients. In practical applications, the choice of method can be made based on the characteristics of the raw materials, cost, and the demands for product purity and activity so as to achieve the desired results.

## 3. The Challenges of Application of DPP-IV Inhibitory Peptides in Food

The incorporation of highly effective DPP-IV inhibitory peptides as functional nutritional ingredients into foods for the prevention or delay of diabetes development in high-risk populations holds significant public health significance [64]. Despite their substantial commercial potential, the application of DPP-IV inhibitory peptides in the food industry still faces numerous challenges that have hindered their commercialization.

### 3.1. The Impact on Food Sensory Perception

The sensory properties of food are critical determinants of consumer acceptance. However, the incorporation of DPP-IV inhibitory peptides may influence the color and flavor profiles of food, particularly introducing bitterness that is often linked to a high content of hydrophobic amino acids in their sequences [27,65]. This can adversely affect the overall sensory attributes of the food [64,66]. To address this issue, researchers have explored various strategies, including adjusting the food formula, adding bitter taste-masking agents, encapsulating peptides, employing filtration or precipitation techniques to eliminate bitter peptides, and utilizing exopeptidases to remove terminal amino acids for debittering [66]. It is worth noting that although the last two methods are effective to some extent, they should be used with caution due to their potential alteration of peptide sequences or concentrations, which could impact biological activity. Moreover, Cermeño et al. [67] reported that cross-linking with transglutaminase, conducted either prior to or following proteolysis, not only clarifies enzymatic hydrolysates, effectively masks bitterness but also preserves ACE inhibitory and DPP-IV inhibitory activities within these enzymatic products. Nevertheless, these findings are based solely on in vitro studies. Therefore, their biological efficacy and stability in vivo require further experimental validation.

### 3.2. Processing Stability

The food processing procedure aims to achieve optimal texture, flavor, stability, and safety of food products. However, this process may inadvertently reduce the stability of DPP-IV inhibitory peptides. The physicochemical stability of peptides is significantly influenced by their sequences, as different amino acid combinations can enhance tolerance to environmental factors [68]. Studies have demonstrated that DPP-IV inhibitory peptides generally exhibit remarkable thermal stability. For example, walnut-derived DPP-IV inhibitory peptides showed only a 5% reduction in activity after exposure to 121 °C for 30 min [24]. Similarly, boarfish protein hydrolysates maintained their DPP-IV inhibitory activity and peptide profiles even after heat treatment at 90 °C for 1 min and 121 °C for 42 s in tomato juice [69].

Nevertheless, in more complex food systems, the stability of peptides may be affected by various factors. The functional groups present in peptides, such as amino, amide, and carboxyl groups, can react with components found in food, including carbohydrates, lipids, phenols, and quinones [70,71]. For example, the Maillard reaction may occur between peptides and reducing sugars under high-temperature conditions. Heating can significantly accelerate this reaction, promote the aggregation of hydrophobic amino acids, and potentially lead to the formation of precipitates. Considering that thermal processing is commonly used in the food industry, it is crucial to strictly control the temperature–time combination to prevent a decrease in the content of target peptides and even the generation of toxic substances such as heterocyclic nitrogen-containing molecules [70].

In order to mitigate the adverse impacts of thermal processing on the nutritional and functional qualities of food, non-thermal processing technologies have garnered significant attention in recent years. Non-heat processing methods, such as ultra-high pressure, ultrasound, microwave, irradiation, and pulsed electric field, can effectively deactivate enzymes and microorganisms at or around room temperature, resulting in minimal damage to the nutritional components of food while achieving improved sensory and nutritional quality [70]. Although non-thermal processing may indirectly affect the stability of peptides; for example, HO· radicals generated by γ radiation can attack peptides. However, antioxidants can partially repair the damage caused by oxidative stress [70]. Currently, there is limited research on the effects of non-thermal processing on DPP-IV inhibitory peptides; further exploration is needed.

The pH value influences the stability of peptides by altering their dissociation state [68]. DPP-IV inhibitory peptides generally exhibit stability across a wide pH range. For example, walnut-derived DPP-IV inhibitory peptides maintain nearly unchanged DPP-IV inhibitory capacity after being treated at room temperature for 30 min within the pH range of 3.0 to 9.0; even following treatment at pH 1.0 or 11.0 for 30 min, the DPP-IV inhibitory activity remains above 90% [24]. López-Sánchez et al. [72] investigated the impact of combined high temperature and pH treatment on the activity of DPP-IV inhibitory peptides. The results indicated that there was no significant difference in the DPP-IV inhibitory activity between amaranth seed protein hydrolysate treated at 100 °C and pH 4.0 for 3 h and the unheated control group. However, after treatment at 120 °C for 1 hour under the same pH value, the DPP-IV inhibitory activity exhibited a significant reduction. This might be attributed to the fact that under the synergistic effects of pH and heat treatment, certain amino acids in the peptide segments are damaged, leading to the denaturation or hydrolysis of specific peptides and consequently influencing their inhibitory activity [72].

Furthermore, the ionic strength of the environment can influence the charge and dissociation of peptides, resulting in alterations to their conformation, solubility, and capacity for molecular interactions [73].

### 3.3. Digestive Stability

To exert their biological activity within the human body, DPP-IV inhibitory peptides must withstand the challenging environment of the gastrointestinal tract after ingestion and effectively cross the epithelial barrier of the small intestine to enter systemic circulation, ultimately reaching their target organs [74,75]. During this process, the digestive stability of DPP-IV inhibitory peptides is crucial. Due to their peptide nature, these inhibitors may undergo various structural modifications or degradation or remain stable under the influence of abundant proteases and peptidases present in the gastrointestinal tract. This depends on their amino acid sequence and length. Consequently, their DPP-IV inhibitory activity may be diminished, enhanced, or unchanged [17,20,25,76].

Nongonierma et al. (2019) [20] reported that, despite the generation of new small peptides during SGID, there were no significant changes in the DPP-IV inhibitory activity of camel whey protein hydrolysate when comparing pre- and post-digestion. Pérez-Gálvez et al. [25] conducted an evaluation of the SGID stability of hydrolysates obtained from various agricultural by-products. The results indicated that both the peptide content and DPP-IV inhibitory capacity of olive seed and sunflower seed hydrolysates remained stable after SGID. In contrast, the peptide content in pea hydrolysate increased following SGID, leading to a significant enhancement in DPP-IV inhibitory activity, as evidenced by a notable reduction in the IC_50_ value. However, despite having a substantial amount of short-chain peptides, the DPP-IV inhibitory activity of lupin hydrolysate decreased after SGID, along with a significant increase in the IC_50_ value. These findings collectively suggest that gastrointestinal digestion introduces considerable uncertainties regarding the activity of DPP-IV inhibitory peptides.

Ahmed et al. [77] conducted a stability analysis of 93 peptide sequences (comprising fewer than 12 amino acid residues) reported in 31 studies for SGID. They found that, in comparison to peptides that exhibited instability during SGID, those resistant to this process displayed smaller molecular sizes, reduced hydrophobicity, a more positive net charge at pH 7.0, an N-terminal branched aliphatic residue, the absence of leucine at the C-terminal end, and elevated levels of His and Pro (with Pro being particularly prominent at the C-terminal) [77].

It is noteworthy that, although the composition of protein hydrolysates is highly complex, it might be only a few high-potential peptide sequences that play a crucial role in their efficacy. In recent years, some studies have increasingly focused on the alterations in both the sequences and activities of these specific peptides after SGID [23,78], which has further enhanced the precision and purposefulness of this research.

### 3.4. Safety Considerations

Although DPP-IV inhibitory peptides hold broad application prospects in functional foods, their commercialization process necessitates a systematic evaluation of safety, regulatory compliance, and market acceptance.

From a safety standpoint, the primary challenges involve assessing the potential allergenicity of food-derived peptides and the toxicological synergy of complex peptide mixtures. For peptides from novel protein sources, dual verification through in vitro allergenicity prediction models and animal experiments is required [79].

Additionally, consumer acceptance is influenced by multiple factors, including the robustness of clinical efficacy evidence, the perception of raw material naturalness, and the demand for clean labels. Notably, non-traditional protein peptides may encounter “novelty rejection”, requiring enhanced market penetration through education and optimization. Achieving coordinated breakthroughs across these three dimensions is critical for industrialization [79].

## 4. The Delivery System of DPP-IV Inhibitory Peptides

### 4.1. The Significance and Ideal Characteristics of the Delivery System

To protect bioactive peptides from adverse environmental factors, thereby preserving their activity and enhancing stability, isolation, and encapsulation through delivery systems are effective strategies. An ideal delivery system for bioactive peptides typically exhibits several key characteristics: Firstly, it should possess superior loading capability (LC) and encapsulation efficiency (EE), facilitating the efficient encapsulation and transport of significant amounts of active peptides. Secondly, it enhances the stability of bioactive peptides, enabling them to resist degradation during prolonged storage and gastrointestinal digestion. Thirdly, it should exhibit targeted release properties that ensure the precise delivery of bioactive substances to specific tissues or cells. Fourthly, it should employ a controlled-release mechanism to regulate the release rate of bioactive substances in response to specific internal signals (such as pH variations or the presence of specific enzymes), thereby prolonging its duration of action within the body. Lastly, possessing good biocompatibility is essential for preventing immune responses or toxicity and minimizing potential side effects.

### 4.2. Common Delivery System Types and Their Effects on DPP-IV Inhibitory Peptides

The delivery of bioactive peptides often hinges on carriers such as gels, liposomes, emulsions, and particles, each characterized by unique attributes. The encapsulation methods of DPP-IV inhibitory peptides and their effects, as reported in the literature, are summarized in Table 3.

Emulsion-based delivery systems capitalize on the immiscibility between oil and water phases to encapsulate DPP-IV inhibitory peptides. High-pressure homogenization and ultrasound are frequently harnessed to fabricate stable emulsions. Puri et al. [80] encapsulated α-lactalbumin-derived hydrolysates. After SGID, the DPP-IV inhibition rate of non-encapsulated hydrolysates was 36%, whereas that of the emulsified counterparts surged to 64%. Animal studies further revealed that the encapsulated form augmented plasma GLP-1 levels by 30% and insulin levels by 25%, underscoring its enhanced capacity to regulate blood glucose. Li et al. [81] crafted a hypoglycemic complex of gallic acid–Antarctic krill polypeptide using polylactic acid-hydroxyacetic acid and high-pressure microjet microencapsulation. Encapsulation extended the half-life of the Antarctic krill polypeptide in simulated gastric juice from 1.5 h to 3.5 h, effectively shielding it from degradation and preserving a higher DPP-IV inhibitory activity.

Liposomes, formed through the self-assembly of phospholipid molecules, serve as efficacious carriers for DPP-IV inhibitory peptides. Wu et al. [75] investigated sodium alginate-coated liposomes harboring DPP-IV inhibition-active collagen peptides. Employing fluorescence-labeled peptide tracking in cell culture, they found that after gastrointestinal digestion, the cell uptake efficiency of peptides in the coated liposomes escalated by 45% compared to non-encapsulated collagen peptides. Montero et al. [82] incorporated nanoliposomes encapsulating an active shrimp peptide fraction into sodium caseinate films. After 10 days of storage at room temperature, the encapsulated shrimp peptide fraction retained 80% of its initial DPP-IV inhibitory activity, while the unencapsulated peptide lost 60% of its activity under identical conditions, vividly demonstrating the protective effect of liposomal encapsulation.

Particle-based delivery systems encapsulate peptides via physical adsorption or interfacial polymerization. Gao et al. [83] prepared bovine serum albumin/chitosan composite nanoparticles for delivering Antarctic krill peptide. These nanoparticles fortified the peptide’s stability. After 4 h of incubation in a simulated intestinal environment, the DPP-IV inhibitory activity of the peptide remained at 70%, while that of the unencapsulated peptide plummeted to 30% within the same period. Wang et al. [84] developed a chitosan and sodium alginate nanocarrier system for rapeseed-derived peptides. In a diabetic mouse model, following 4 weeks of treatment, the encapsulated rapeseed-derived peptides decreased blood glucose levels by 35%, while the non-encapsulated peptides achieved only a 15% reduction, manifesting the enhanced anti-diabetes therapeutic efficacy of the nanocarrier-encapsulated peptides.

Hydrogel-based delivery systems encase DPP-IV inhibitory peptides within a three-dimensional polymer network. Pugliese et al. [85] developed novel soybean and lupin peptide nanogels. In-vitro enzyme inhibition assays determined that the DPP-IV inhibitory activity of the encapsulated peptides increased by 2.5-fold relative to the free peptides. Lammi et al. [86] utilized self-assembling peptide-based hydrogels to encapsulate hempseed hydrolysates. After 24 h incubation in human serum, the hydrogel-encapsulated hempseed hydrolysates maintained 75% of their anti-DPPIV activity, while the unencapsulated ones retained merely 30% of their activity, evidencing the substantial stability-enhancing effect of hydrogel encapsulation.

**Table 3:** The properties and effects of encapsulation of DPP-IV inhibitory peptides and hydrolysates in the literature.

Source of DPP-IV Inhibitory Peptides	Carrier Type	Encapsulation Materials	Preparation Approaches	Property Characterization	Stability and Efficacy	References
α-lactalbumin hydrolysates	W1/O/W2 double emulsion	Polyglycerol polyricinoleate in rice bran oil and pectin	Homogenization	Particle size: 17.33 ± 0.97 μm Zeta potential: −35.43 ± 1.85 mV EE: 93.00 ± 0.90%	Stable for 90 days at 5 ± 1 °C and 20 days at 37 ± 1 °C DPP-IV inhibition: 36% (non-encapsulated), 54% (freeze-dried encapsulated), 64% (emulsified encapsulated hydrolysates) after SGID Reduced blood glucose Increased plasma GLP-1 and insulin levels Normalized lipid profile, atherogenic index, catalase/SOD activity, liver enzymes (ALT, AST, AP)	[80]
Brewer’s spent grain hydrolysate	Microcapsules	Carrageenan, agar, maltodextrin	Spray drying	Particle size: 1.5 to 25 μm Zeta potential: negative at pH 2.0 and 7.0 EE: 34.14% to 50.28%	Enhanced bioaccessibility of peptides with antioxidant and antihypertensive activity Higher peptide release at pH 7.0 than at pH 2.0. High protection of antioxidant, ACE-I inhibitory, and DPP-IV inhibitory activities after SGID.	[87]
Lupin peptide and soybean peptide	Nanogels	An ionic self-assembling peptide RADA16	Solvent-triggered co-assembly	Soft hydrogel profiles Shear-thinning, injectability Stable cross-β sheets	Increased protease resistance Enhanced DPP-IV inhibition in Caco-2 cells Delayed peptide release	[85]
Spirulina protein hydrolysates	Microcapsules	Alginate and chitosan	Extrusion	EE: 86.30% ± 0.16% LC: 80.65% ± 0.16%	Minimal release in simulated gastric fluid Gradually release in simulated intestinal fluid Retained DPP-IV inhibitory activity	[88]
Tenebrio molitor hydrolysate	Nano-microcapsules	Arabic gum, pullulan, Tween 20	Electrospraying, spray-drying	Electrosprayed: smaller size (1.2 ± 0.5 µm), uniform; lower surface nitrogen with additives (better encapsulation) Spray-dried: larger size (12.4 ± 8.7 µm with additives, 11.3 ± 5.76 µm without additives), less uniform; higher surface nitrogen without additives (potential peptide degradation)	Initial: IC_50_ = 1.29 ± 0.07 mg protein/mL Electrosprayed: IC_50_ = 1.50 ± 0.07 mg protein/mL (16.16% decrease) Spray-dried (with additives): IC_50_ = 1.61 ± 0.08 mg protein/mL (24.13% decrease) Spray-dried (without additives): IC_50_ = 1.99 ± 0.03 mg protein/mL (53.60% decrease)	[89]
Hempseed protein hydrolysates	Hydrogel	RADA16 self-assembling peptide	Self-assembly	Nanofibril network (about 24 nm diameter, up to 2 μm length), β-sheet signatures Increased G’ values	Enhanced stability in human serum Synergistic DPP-IV inhibition with sitagliptin	[86]
Collagen peptides	Liposomes	Lecithin from soybean, sodium alginate	Coacervation coating	Sodium alginate-coated collagen peptide liposomes: more increased particle size, polydispersity index (PDI), absolute zeta potential, and EE compared to collagen peptide liposomes	Collagen peptide liposomes: higher release rate in SGID, lower stability at 4 °C in the dark for 40 days Sodium alginate-coated collagen peptide liposomes: lower release rate in SGID, higher stability at 4 °C in the dark for 40 days, enhanced cellular uptake and secretion in Caco-2/HT29 cell model, improved penetration through mucus layer	[75]
Phaseolus lunatus seed protein hydrolysates	Microcapsules	Maltodextrin and gum arabic	Spray drying	Increased yield, protein efficiency, and protein release at pH 7.0	Protected inhibitory activities on α-glucosidase, α-amylase, and DPP-IV Minimal protein release in gastric fluid Partial protection from digestive enzymes for ACE-I inhibitory activity	[90]
Brewer’s spent grain-hydrolysate	Microcapsules	Agar and maltodextrin	Spray drying	(Not explicitly mentioned in the document)	Reduced serum glucose Inhibited intestinal α-glucosidase Reduced cecal α-amylase activity Inhibited serum and intestinal DPP-IV	[91]
Goat milk whey protein peptide (MW < 3 kDa)	Liposomes (GWP-LS) and niosomes (GWP-NS).	Lecithin, phytosterols (β-sitosterol, ergosterol, stigmasterol, mixed phytosterols)	Ethanol injection, stirring, and rotary evaporation	Higher EE (90.46 ± 4.02% for GWP-NS) Smaller particle size (92.07 ± 9.8 nm for GWP-NS)	Improved SGID stability (GWP-NS) Increased GWP bioaccessibility Thermal stability improvement (25 °C, 37 °C) Hypoglycemic activity: not affected by nanoencapsulation, improved after loading (GWP-NS) Masked bitter taste of GWP	[92]
Gallic acid-Antarctic krill peptide copolymer	Nanocapsules	Polylactic acid-hydroxyacetic acid	Complex emulsion method, high-pressure microjet	Uniformly embedded in capsules Particle size: 300 to 400 nm Smooth and dense surface	Reduces diffusion in stomach, increases drug reaching intestine Improves thermal and hygroscopic stability Effective inhibition of α-amylase, α-glucosidase, and DPP-IV at different storage temperatures	[81]
Antarctic krill peptide	Composite nanoparticles	Bovine serum albumin and chitosan	Ionotropic gelation and coacervation	Particle size: 83.3 ± 4.4 to 222.4 ± 32.7 nm Zeta-potential: 35.1 ± 0.7 to 45.0 ± 2.7 mV PDI: 1.000 ± 0.002 to 0.306 ± 0.011 Morphology: spherical, uniform, tightly bound	Excellent stability in pH 2–5 Stable after 15 days Hindered release in gastric environment Promoted release in intestinal environment Retained hypoglycemic activity	[83]
Rapeseed-derived cruciferin peptide (RCPP) and napin peptide (RNPP)	Nanoparticles	Chitosan and sodium alginate	Three-channel device	Particle size: 141.8 ± 1.75 nm (CS/ALG-RNPP), 220.2 ± 2.11 nm (CS/ALG-RCPP) Zeta potential: -40.27 mV (CS/ALG-RNPP), −39.75 mV (CS/ALG-RCPP) EE: 90.7% (CS/ALG-RNPP), 91.4% (CS/ALG-RCPP) LC: 15.38% (CS/ALG-RNPP), 16.63% (CS/ALG-RCPP)	Significant sustained-release effect in simulated intestinal fluid Three-fold increase in GLP-1 secretion compared to RPP groups Enhanced expression of calcium-sensing receptor in CS/ALG-RCPP	[84]
Low MW peptide fraction from a shrimp hydrolysate	Nanoliposomes	Partially purified soy phosphatidylcholine	Stirring and ultrasonication	Particle size: 99.98 ± 4.0 nm PDI: 0.186 ± 0.006 Zeta-potential: −53.86 ± 2.91 mV EE: 52.37 ± 2.38%	Films with liposomes had favorable taste perception and faster buccal dissolution No signs of bitterness or astringency Increased water solubility, adhesion, and mucoadhesion Improved thermal and hygroscopic stability of peptides	[82]
Whey protein hydrolysate	Micro-hydrogels	Chitosan and gelatin	Spray drying	(Not explicitly mentioned in the document)	Chitosan encapsulation: no change in peptide profile; better degradation protection (5/21 peptides protected) Gelatin: complex composition, difficult peptide identification; 1/21 peptides protected	[93]
Rapeseed peptides	Nanogel	RADA16	Self-assembly gelation	Stable β-sheet structures increased above 5-fold Self-assembled fibrous morphology	Significant DPP-IV inhibitory activity enhancement (>65%) GLP-1 secretion increase (35.46%) Increased intracellular calcium ion mobilization Increased cAMP concentration High dispersibility in water Significant sustained-release effect Enhanced thermal and hygroscopic stability	[94]

### 4.3. Selection and Optimization of the Delivery System

Rational design is required for the delivery carrier types employed. The appropriate type of delivery carrier should be determined based on factors, including the physicochemical properties of the peptide (e.g., solubility, charge characteristics, stability, molecular size), the type of target food, processing conditions (e.g., pH value, temperature, pressure, processing duration), and anticipated shelf life [95]. For example, hydrophilic DPP-IV inhibitory peptides are suitable for hydrophilic carriers, such as certain hydrogels, while hydrophobic peptides are more suitable for hydrophobic carriers like liposomes. The encapsulation efficiency, stability, and release kinetics of DPP-IV inhibitory peptides are influenced by various interactions between the peptides and wall materials, including electrostatic interactions, hydrophobic interactions, hydrogen bonding, van der Waals forces, and covalent bonding [96]. Stronger interactions, such as covalent bonding, tend to enhance stability but may impede peptide release, while weaker interactions, such as van der Waals forces, promote faster release. Adjusting parameters such as pH, temperature, or ionic strength can dynamically regulate these interactions, thereby enabling targeted delivery.

The materials utilized for the encapsulation and delivery of bioactive peptides typically refer to natural macromolecular substances such as proteins, polysaccharides, or lipids [97].

The materials employed tend to exert a remarkable influence on the stability and release characteristics of the delivery system. For example, Gómez-Mascaraque et al. [93] encapsulated whey protein hydrolysate using chitosan and gelatin as wall materials. After SGID, there was a reduction in the quantity of free hydrolyzed peptides, while the peptide profile within chitosan microcapsules showed minimal changes. In contrast, the chromatogram of digestive products in gelatin capsules was complex and difficult to interpret. During the yogurt fermentation experiment, five peptides were effectively protected within chitosan microcapsules, while only one peptide remained in those utilizing gelatin as the wall material. Therefore, chitosan is deemed more suitable than gelatin for serving as a delivery carrier in preparing such peptides [93].

To attain the desired delivery efficacy, researchers frequently combine diverse approaches for carrier fabrication. For instance, polysaccharides like chitosan and sodium alginate possess significant mucosal adhesiveness [98,99,100]. Consequently, many researchers apply polysaccharide coatings on the basis of traditional emulsions or liposomes, with the aim of enhancing the targeting property of the delivery system and the bioavailability of active substances. Wu et al. [101] developed liposomes encapsulating DPP-IV inhibitory peptides derived from sheepskin and subsequently applied a sodium alginate coating. The results indicated that liposomes coated with sodium alginate demonstrated greater stability in both SGID and 40-day storage experiments and improved the transcellular permeability of collagen peptides.

Similarly, innovative systems for the delivery of bioactive proteins and peptides have been established through the integration of various delivery carrier technologies, including liposomes and emulsions, emulsions and gels, emulsions and particles, as well as liposomes and particles [102]. This integration approach significantly enhances the design flexibility, facilitating customization according to specific application scenarios. Such a strategy contributes to improving key performance metrics of delivery carriers, including stability, bioavailability, and targeting, thereby providing a more effective solution for the successful delivery of bioactive proteins and peptides. Despite the potential exhibited by these methods, their implementation in DPP-IV inhibitory peptide delivery remains limited; thus, further research is necessary to validate their efficacy and feasibility.

## 5. Prospects

### 5.1. The Influence of Encapsulation on Food Sensory Perception

The design of delivery carrier applications in food necessitates careful consideration, as their encapsulation forms and specific physicochemical properties can significantly influence the sensory quality of the product. For example, the system typically exhibits high clarity when the particle size is below 50 nm. As the particle size increases gradually, turbidity correspondingly rises [103]. Especially when particle sizes approach the millimeter level, such as some hydrogel beads, these particles become visible to the naked eye, which undoubtedly impacts the sensory attributes of the food. Additionally, particle size affects mouthfeel. To alleviate coarse or unpleasant textures for consumers caused by larger particles, it is typically necessary to maintain the particle size of the delivery carriers at a relatively low level. Nevertheless, particle size is not the sole determining factor influencing the gritty perception. The quantity of particles and the viscosity of the system also exert an influence on the human body’s perception of the particles [104].

### 5.2. Safety

While it is widely accepted that bioactive peptides are safe for human consumption, potential allergenicity and toxicity warrant careful consideration, particularly in cases of high-dose administration and long-term intake. Therefore, further in vitro and in vivo toxicity studies are essential, especially for the safety of other substances introduced through encapsulation. Microemulsions, due to their high stability and good encapsulation performance, are frequently employed as delivery carriers for peptides. However, the preparation of microemulsions requires a substantial amount of surfactants, and there is a limited selection of reliable food-grade emulsifiers [105]. Prolonged consumption of these surfactants may bring about health risks, which contradicts the original intention of promoting health.

### 5.3. Co-Encapsulation

Research on the encapsulation of DPP-IV inhibitory peptides can benefit from the successful experiences of other peptide studies. A promising strategy involves co-encapsulating active substances with synergistic effects alongside DPP-IV inhibitory peptides and targeting release, thereby enhancing bioavailability and efficacy. For example, DPP-IV inhibitory peptides could be co-encapsulated with other active compounds that have been demonstrated to be beneficial for diabetes prevention and treatment, such as α-glucosidase inhibitors, specific polysaccharides, flavonoids, or polyphenols. By investigating the interactions between DPP-IV inhibitory peptides and these additional active substances, candidates exhibiting synergistic effects can be identified for subsequent co-encapsulation to amplify overall effectiveness. Furthermore, diabetic patients not only experience impaired glucose metabolism but often also exhibit other metabolic abnormalities, including hyperlipidemia and increased oxidative stress [106]. Consequently, when developing a product, it may be essential to consider the combined use of complementary components or seek out multifunctional DPP-IV inhibitory peptides, such as those possessing anti-hyperglycemic, antioxidant, and lipid-lowering properties, to better address the comprehensive needs of diabetic patients.

### 5.4. Targeted Delivery

While bioactive peptides exhibit relatively minor side effects, their efficacy is generally inferior to that of pharmaceuticals. Therefore, to enhance the bioavailability of bioactive peptides, targeted delivery is necessary. Furthermore, the stability of the delivery system demands rigorous control and validation to avoid premature or delayed release, as these would significantly compromise the effectiveness of peptide applications.

### 5.5. Scalable Approaches

Developing scalable and cost-effective methods for the preparation, purification, and encapsulation of DPP-IV inhibitory peptides is crucial. This involves refining existing techniques and exploring new technologies to ensure high yields and consistent quality. While there are many reports on large-scale production concerning peptide preparation and purification, relatively few such reports exist in the domain of encapsulation. For example, the technique for preparing gel beads via dripping encounters significant challenges for large-scale application. To address these limitations, alternative scalable techniques such as extrusion and spray drying can be explored [107]. These methods offer consistent production and enhanced scalability, ensuring high-quality encapsulation without compromising the integrity of the peptides.

By adopting these advanced techniques, it is possible to achieve scalable production processes that maintain the functional properties of the encapsulated peptides, thereby facilitating their broader application in pharmaceuticals and functional foods.

### 5.6. The Application Effect in Food

While numerous studies have explored the potential challenges associated with using unencapsulated DPP-IV inhibitory peptides in food applications and the stabilizing effects of encapsulation technology, a notable gap remains in understanding how encapsulated peptides affect the sensory properties of food and their variations during processing and storage. Therefore, it is imperative to conduct in-depth research on precise delivery strategies and the practical application effects of these delivery systems in different types of food matrices. Consumer acceptance studies can provide valuable insights into the market potential of these functional foods.

## 6. Conclusions

This article provides a comprehensive summary of the advancements and challenges associated with the primary strategies for preparing DPP-IV inhibitory peptides (including enzymatic hydrolysis, fermentation, and synthesis) as well as encapsulation technologies (such as emulsions, microcapsules, nano-gels, etc.). It highlights the critical barriers in sensory characteristics, processing/digestive stability, bioavailability, large-scale production, and safety/regulatory compliance of DPP-IV inhibitory peptides. Furthermore, it outlines more specific and targeted future research directions, such as the development of multifunctional peptides, co-encapsulation techniques, exploration of targeted delivery systems, evaluation of application efficacy in real food matrices, enhancement of safety assessments, and promotion of standardization.

In conclusion, this review underscores the significant progress in the development of preparation techniques for DPP-IV inhibitory peptides, emphasizing their potential as functional ingredients in the food industry. Future research should focus on developing cost-effective and scalable technologies for the preparation of by-product peptides while investigating their stability and compatibility within genuine food systems. By addressing these key areas, we can pave the way for the commercialization of DPP-IV inhibitory peptides, making them viable, functional food ingredients that offer tangible health benefits to consumers.

## Figures and Tables

**Table 1:** Comparison of Preparation Methods for DPP-IV Inhibitory Peptides.

Method	Advantages	Disadvantages	Food Applicability
Enzymatic Hydrolysis	Mild conditions Safe and environmentally friendly Scalable	Limited specificity Potential bitterness High cost of proteases Incomplete hydrolysis	High (aligned with clean-label trends)
Fermentation	Natural Rich flavor Cost-effective	Time-consuming Low yield Poor specificity Complex composition	Moderate (depends on strain safety)
Chemical Synthesis	High purity Sequence control	High cost Synthetic origin	Low (limited to research validation)
Enzymatic Synthesis	Mild conditions Food-grade materials Scalable	Enzyme selection Optimization complex High cost and liability of enzymes	Moderate (suitable for specific peptides)

**Table 2:** Source, Sequence, and Inhibitory Activity of Food-Derived DPP-IV Inhibitory Peptides.

Source	Preparation Method	IC_50_ of Hydrolysate (mg/mL)	Peptide Sequence	Size (AA)	IC_50_ of Peptides (µM)	Reference
ENZYMOLYSIS						
Oat protein isolate	Alcalase	0.41	SPVAEVPFLR	10	167.8	[29]
			LDATDMVALVG	11	269.1	
Camel milk proteins	Trypsin	0.52	LPVP	4	87	[30]
			MPVQA	5	93.3	
			YPVEPF	6	138	
			LLQLEAIR	8	177.8	
			SPVVPF	6	214.1	
			ILDKVGINY	9	321.5	
			ILDKEGIDY	9	347.8	
			ILELA	5	721.1	
Chicken blood	Alcalase and Protana Prime		GPF	3	940	[21]
			IGL	3	2220	
			GGGW	4	2730	
*Musculus senhousei*	Neutrase		DPF	3	1399.73	[31]
			LTWR	4	1788.67	
Bactrian camel milk	α-Chymotrypsin and Proteinase K		QPY	3	655.60	[32]
			FPH	3	1039.29	
			LPAAP	5	199.66	
			WPEYL	5	380.16	
			YPPQVM	6	1067.49	
			IPAPSFPRL	9	425.01	
Discarded cowhide collagen	Compound protease and Papain	3.04	GPVG	4	386.77	[33]
			FGPGP	5	3309.21	
			APGGAP	6	382.07	
			GPPGPT	6	1197.14	
			GPVGPPG	7	196.67	
Coix seed prolamins	Papain and Alcalase		LPFYPN	6	70.24	[34]
			TFFPQ	5	176.87	
			ATFFPQ	6	268.31	
Tilapia skin gelatin	Ginger protease		GPXGPPGPGP	9	1012.3	[18]
Boarfish (*Capros aper*)	Alcalase 2.4L and Flavourzyme 500L		IPV	3	5.61	[17]
			APIT	4	34.73	
			VPTP	4	38.93	
			GPIN	4	48.96	
			IPGA	4	66.37	
			GPSL	4	68.13	
			GPSI	4	72.85	
			APVP	4	73.15	
			APLT	4	91.1	
			MPAVP	4	115.27	
			GPGI	4	116.27	
			GPLN	4	116.37	
			PAVP	4	126.51	
			GPGL	4	131.9	
			LPGA	4	154.12	
			AALP	4	164.37	
			IPVDM	5	21.72	
			LPVYD	5	51.36	
			LPVDM	5	53.5	
			APLER	5	63.67	
			VPDPR	5	79.1	
			APLDK	5	90.37	
goat milk whey protein	Papain	0.34	FNPTY	5	62.32	[35]
			LDADGSY	7	52.16	
			SPPEFLR	7	56.22	
			YPVEPFT	7	175.7	
FERMENTATION						
fermented Mandarin fish (*Chouguiyu*)	*Lysobacter*, *Lactococcus*		GEKVDFDDIQK	11	-	[36]
	*Lysobacter*, *Lactococcus*		VVDADEMYLKGK	12	-	
	*Lactococcus*, *Peptostreptococcus*		GQKDSYVGDEAQ	12	-	
	*Bacillus*, *Kocuria*		KAGARALTDAETAT	14	-	
cow milk	*Limosilactobacillus fermentum*	0.18				[37]
camel milk	*Limosilactobacillus fermentum*	0.37				
goat milk	*Limosilactobacillus fermentum*	0.36				
sheep milk	*Limosilactobacillus fermentum*	0.26				
whey protein concentrate	*Streptococcus thermophilus*		IPA	3	49	[38]
			IPP	3	169	
			LPVP	4	87	
			VLGP	4	580	
			VPYPQ	5	41	
			LPVPQ	5	44	
			APFPE	5	49	
			YPFPGP	6	749	
			PQNIPPL	7	1500	
			TPEVDDEALEK	11	320	

## Data Availability

No new data were created or analyzed in this study. Data sharing is not applicable to this article.

## Abbreviations

The following abbreviations are used in this manuscript:

DPP-IV	Dipeptidyl peptidase IV
GLP-1	Glucagon-like peptide-1
SGID	Simulated gastrointestinal digestion
LC	Loading capacity
EE	Encapsulation efficiency
MW	Molecular weight
PDI	Polydispersity index
